# Inter-Laboratory Assessment of a Prototype Multiplex Kit for Determination of Recent HIV-1 Infection

**DOI:** 10.1371/journal.pone.0077765

**Published:** 2013-10-17

**Authors:** Kelly A. Curtis, Andrew F. Longosz, M. Susan Kennedy, Sheila Keating, John Heitman, Oliver Laeyendecker, S. Michele Owen

**Affiliations:** 1 Laboratory Branch, Division of HIV/AIDS Prevention, National Center for HIV/AIDS, Hepatitis, STD, and TB Prevention, Centers for Disease Control and Prevention, Atlanta, Georgia, United States of America; 2 Laboratory of Immunoregulation, Division of Intramural Research, National Institute of Allergy and Infectious Diseases, NIH, Baltimore, Maryland, United States of America; 3 Blood Systems Research Institute, San Francisco, California, United States of America; 4 Department of Medicine, Johns Hopkins University, Baltimore, Maryland, United States of America; Istituto Superiore di Sanità, Italy

## Abstract

**Background:**

Accurate and reliable laboratory-based assays are needed for estimating HIV-1 incidence from cross-sectional samples. We recently described the development of a customized, HIV-1-specific Bio-Plex assay that allows for the measurement of HIV-specific antibody levels and avidity to multiple analytes for improved HIV-1 incidence estimates.

**Methods:**

To assess intra- and inter-laboratory assay performance, prototype multiplex kits were developed and evaluated by three distinct laboratories. Longitudinal seroconversion specimens were tested in parallel by each laboratory and kit performance was compared to that of an in-house assay. Additionally, the ability of the kit to distinguish recent from long-term HIV-1 infection, as compared to the in-house assay, was determined by comparing the reactivity of known recent (infected <6 months) and long-term (infected >12 months) drug naïve specimens.

**Results:**

Although the range of reactivity for each analyte varied between the prototype kit and in-house assay, a measurable distinction in reactivity between recent and long-term specimens was observed with both assays in all three laboratories. Additionally, kit performance was consistent between all three laboratories. The intra-assay coefficient of variation (CV), between sample replicates for all laboratories, ranged from 0.5% to 6.1%. The inter-laboratory CVs ranged from 8.5% to 21.3% for gp160-avidity index (a) and gp120-normalized mean fluorescent intensity (MFI) value (n), respectively.

**Conclusion:**

We demonstrate the feasibility of producing a multiplex kit for measuring HIV antibody levels and avidity, with the potential for improved incidence estimates based on multi-analyte algorithms. The availability of a commercial kit will facilitate the transfer of technology among diverse laboratories for widespread assay use.

## Introduction

Since the use of a serologic assay for estimating HIV-1 incidence from cross-sectional samples was first described in 1998 [1], considerable emphasis has been placed on the development and optimization of laboratory techniques for distinguishing recent from long-term HIV infection. Accurate methods of estimating HIV incidence are essential for monitoring trends in transmission, identifying high-risk populations, and evaluating the efficacy of prevention measures [2]. Typically, tests for recent infection (TRIs) have involved the measurement of HIV-specific antibody responses or avidity, based on the increasing pattern of antibody reactivity observed in HIV-1 infected individuals post-seroconversion [3,4]. To enhance the predictive value between samples from recent and long-term infected individuals, serologic assay approaches have included the modification of commercial kits, to reduce the sensitivity of detection [1,5,6], or a dissociation step [7,8], as a measure of antibody avidity. In the absence of manufacturer support, off-label use of commercial diagnostic tests may be problematic, especially in disseminating a common protocol to all potential users and relying on continued production. Several in-house serologic assays have also been developed, specifically for the purpose of determining recent HIV-1 infection [9-12]. For widespread use, in-house techniques must be validated to assess intra/inter-laboratory assay performance prior to large-scale production.

The BED capture immunoassay (BED-CEIA), the most widely used assay designed solely for the purpose of estimating HIV incidence, measures the proportion of antibody directed against an immunodominant branched gp41 peptide [9,13]. The intra- and inter-laboratory variability of the BED-CEIA assay has been described in detail by Dobbs et al. [14]. The BED-CEIA assay was the first TRI to become commercialized and has been used worldwide for HIV-1 incidence surveillance purposes [15-18]. Despite its widespread availability, the assay has undergone scrutiny based on reports describing high false-recent rate (FRRs) in some populations, which can lead to the overestimation of HIV incidence [19-22]. Recently, the HIV-1 Limiting Antigen (LAg)-Avidity EIA, which measures binding of high-avidity antibodies to a subtype-conserved, recombinant gp41 protein, has been commercialized for HIV-1 surveillance use [10,23]. Given the public health implications of inaccurate incidence estimates, new and developing TRIs must be comprehensively validated prior to their implementation. We have described the development of an in-house, HIV-1-specific Bio-Plex assay for determining recent HIV-1 infection [11]. The multiplex assay measures both HIV-specific antibody levels and avidity to multiple analytes in a single 96-well assay plate. Recent studies have shown the FRR can be eliminated or reduced when using a multi-test algorithm approach for determining recent infection [24,25]. Furthermore, improved HIV-1 incidence estimates have been demonstrated using algorithms of multiple analyte measures obtained from the HIV Bio-Plex assay [26].

Sine initial proof-of-concept studies with the in-house multiplex assay have yielded promising results; we investigated the feasibility of developing a kit for widespread dissemination and use. We evaluated a prototype multiplex kit to measure HIV-1-specific antibody levels and avidity. The performance of the kits and intra-assay variation were compared to our in-house methods [11]. Inter-laboratory performance was assessed amongst three participating laboratories. These results will aid in determining the practicality of developing a bead-based multiplex kit for estimating HIV incidence and will enable further development of the assay, with the intent of producing a consistent product for widespread use. 

## Materials and Methods

### In-house HIV-1-specific Bio-Plex assay

The HIV-1-specific Bio-Plex assay was performed in the reference laboratory (laboratory 1) as previously described [11], with the exception that magnetic COOH beads (Bio-Rad Laboratories, Hercules, CA) were used for the antigen coupling procedure as opposed to polystyrene beads used in earlier studies. The beads were coupled with recombinant HIV-1 gp120, gp160, and gp41 proteins (Immunodiagnostics, Inc., Woburn, MA) for detection of HIV-1-specific IgG levels and avidity. All plasma samples were tested in duplicate. A normalized mean fluorescent intensity (MFI) value was calculated relative to the MFI of the calibrator and avidity index (AI) was determined as described [11]. 

### HIV-1-specific multiplex kits

Magnetic bead-based multiplex kits were designed and produced by Radix BioSolutions, Ltd. (Georgetown, TX), according to the methods described previously [11]. To conserve costs, the bead mix was composed of bead sets specific for three HIV-1 analytes: gp120, gp160, and gp41. The HIV envelope proteins were selected for inclusion in the kits, as they provide the greatest measurable distinction between recent and long-term samples [11]. Additionally, anti-IgG and bovine serum albumin (BSA)-specific bead sets were included in the bead mix for sample loading and non-specific binding controls, respectively. Each kit contained enough reagents for a 96-well test plate. Specific components included: 10X bead mix, sample dilution buffer [11], assay buffer [11], 0.1M DEA in assay buffer, and detection antibody (PE-conjugated, goat anti-human IgG). Each of the three participating laboratories evaluated 5 kits from the same production lot. Additionally, a second lot of kits was produced to assess lot-to-lot variation, which consisted of 3 kits per laboratory. Each laboratory was provided with the same calibrator used for the in-house procedure. If the MFI of the IgG control bead was not within 25% of the duplicate for a particular specimen, the lower value was considered invalid and that replicate was not included in subsequent analyses. 

### Specimens

To assess variation between the in-house assay versus the prototype kits and between three different laboratories, 114 longitudinal specimens from 21 recent seroconverters were tested in parallel. The specimens had previously been collected as part of the Vaccine Preparedness Study for The HIV Network for Prevention Trials (HIVNET), as described [11,27,28]. The sample number was chosen based upon the availability of the kits and the number of specimens that could be tested per kit. Of the 114 specimens tested, 43 were collected at time points when the study subject was on ART, based on self-report. An estimated date of seroconversion was determined by calculating the midpoint between the last negative antibody assay and the first positive Western blot test date. The interval of time between the last negative and first positive antibody test ranged from 71-202 days. To evaluate kit performance, reactivity in anti-retroviral (ARV)-naïve samples from individuals with known recent (n=27) or long-term (n=23) infection was compared. Samples were defined as recent if collected <6 months post-seroconversion and long-term if collected >12 months post-seroconversion. To evaluate the second lot of kits, 69 ART-naïve HIVNET specimens were repeat tested. 

### Statistical analysis

Differences in normalized MFI and avidity index values between known recent and long-term specimens were evaluated using the Wilcoxon rank sum test. 

## Results

### Comparison of in-house assay to prototype kit

The MFI values for the calibrator were similar between our in-house HIV-1-specific Bio-Plex assay and the prototype kit for gp41; however, the kit values for gp160 and gp120 were approximately half that of the in-house assay (Table 1). The minimum and maximum values, along with the 25^th^-75^th^ percentile of reactivity, for 114 longitudinal seroconversion specimens are shown in Table 1 for the following analytes: gp160-normalized MFI value (n), gp41-n, gp120-n, gp160-avidity index (a), gp41-a, and gp120-a. The kit exhibited a greater range of reactivity for gp160-n and gp120-n, as indicated by a higher maximum value and 25^th^-75^th^ percentile of reactivity. The range of reactivity was similar for the avidity values. The correlation coefficient, evaluating concordance between the two assays for all 6 analytes, ranged from 0.83 to 0.96 (Table 1). 

**Table 1:** Comparison of in-house, HIV-1-specific Bio-Plex assay and prototype kit.

**Analyte**	**Calibrator^a^ (SD)**			**Min, Max Reactivity (25%-75%)**		**Correlation Coefficient (r)**
	**In-house**	**Kit**		**In-house**	**Kit**	
gp160-n	8563 (1129)	4283 (278)		0.4, 3.1 (2.1-2.8)	0.3, 6.2 (3.3-5.4)	0.94
gp41-n	20066 (1588)	18078 (450)		0.8, 1.4 (1.2-1.3)	0.7, 1.5 (1.3-1.4)	0.83
gp120-n	2348 (452)	1363 (131)		0.2, 7.8 (2.4-5.4)	0.2, 14.3 (2.5-7.4)	0.95
gp160-a	ND	ND		4.1, 90.6 (38.7-63.7)	4.0, 90.0 (29.1-50.8)	0.91
gp41-a	ND	ND		7.2, 93.6 (36.2-70.9)	4.7, 96.9 (30.7-69.2)	0.96
gp120-a	ND	ND		0.0, 77.3 (14.0-39.6)	0.8, 76.1 (11.0-29.8)	0.88

^a^Mean MFI value from 5 separate assay plates.

**Figure 1:**
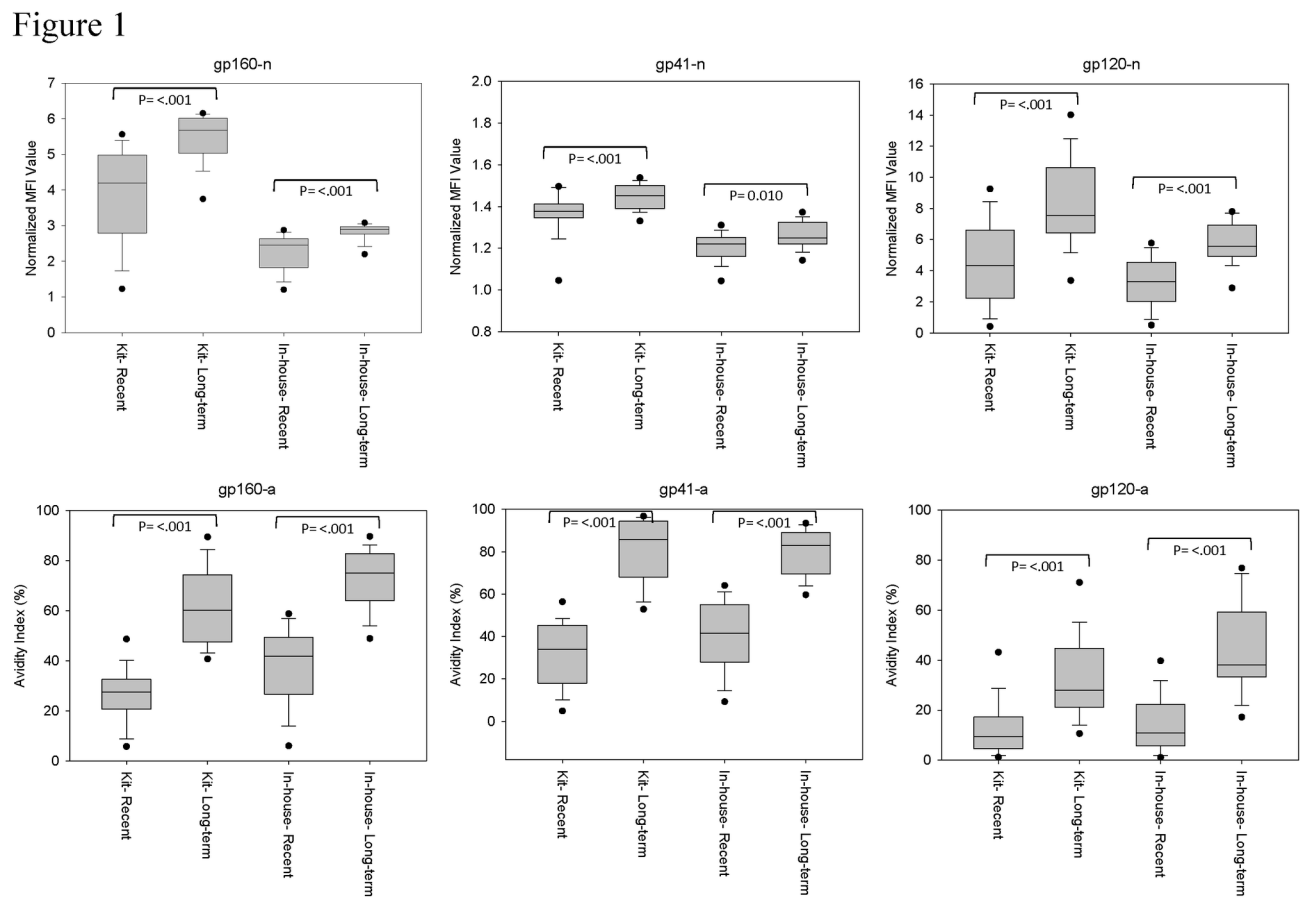
Performance of in-house assay versus prototype kit. The normalized MFI values (n) and avidity index (a) for the in-house assay and kit were compared for drug naïve, known recent (infected <6 months) and long-term (infected >1 year) specimens. The boxes represent the 25^th^ to 75^th^ percentile of reactivity, while the middle lines represent the median values. Black dots indicate the 5^th^ to 95^th^ percentile of reactivity.

To assess the ability of the kit to distinguish recent from long-term HIV-1 infection, as compared to the in-house assay, the normalized values and avidity indexes of known recent and long-term ARV-naïve specimens were compared (Figure 1). Although the range of reactivity differed between the in-house assay and kit, there was a significant difference between recent and long-term specimens for all analytes. With the exception of gp41-n, there was minimal to no overlap between 25^th^-75^th^ percentile of normalized MFI values and avidity index for recent and long-term specimens. Discrimination in assay results between recent and long-term specimens was greater with the avidity measures, relative to the normalized values, for both assays. 

### Reproducibility of kit performance

**Figure 2:**
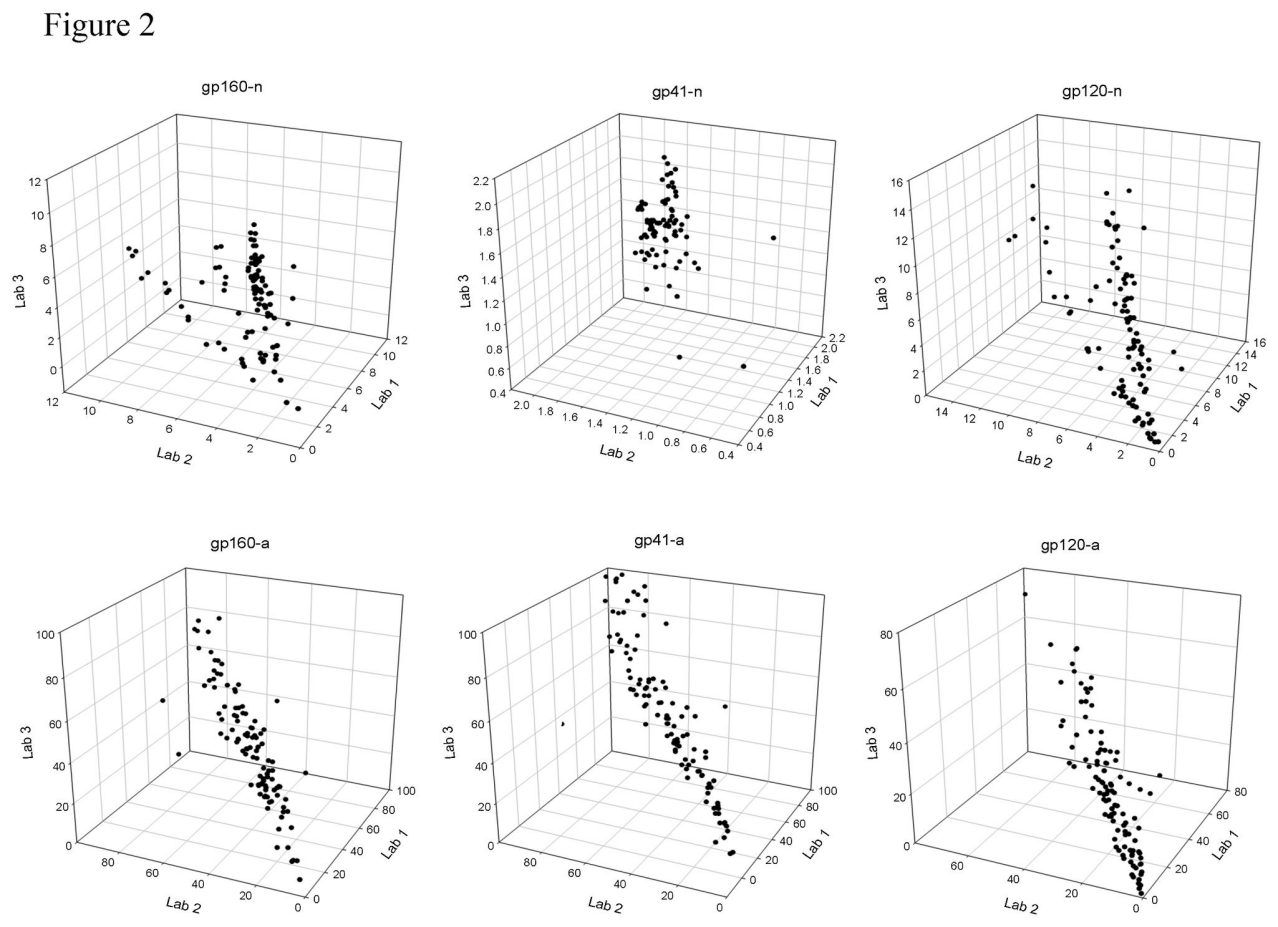
Inter-laboratory reproducibility of kit measures. The normalized MFI values and avidity index of longitudinal seroconversion panels were compared for the three participating laboratories. The values for each laboratory were plotted on a separate axis.

Intra- and inter-assay reproducibility of the kits was evaluated by comparing the assay results of the longitudinal seroconversion panels from three laboratories (Table 2). The median intra-assay coefficients of variation (CVs) for each analyte were similar between the three laboratories and showed consistent performance between sample replicates for all analytes. The median intra-assay CVs for all laboratories ranged from 0.5% for gp41-n to 6.1% for gp120-a. The correlation coefficient (r) between each laboratory was ≥0.85 for all avidity measures. Inter-laboratory concordance was variable for the normalized MFI values, depending on the specific analyte. For gp160-n, laboratory 2 produced assay results that deviated from the other two laboratories, yielding *r* values of 0.49 and 0.39 as compared to 0.92 for laboratory 1 versus 3. The gp41-n exhibited the lowest *r* values of -0.05, 0.60, and -0.13. The median inter-laboratory CVs for all three laboratories ranged from 9.9% for gp41-n to 21.3% for gp120-n. Normalized MFI values and avidity index of each sample tested are shown in Figure 2 for all three laboratories. A comparison of kit lots 1 and 2 yielded CVs ranging from 5.0% for gp41-n to 18.4% for gp120 (Table 3).

**Table 2:** Intra-assay and inter-laboratory performance of prototype kit.

**Analyte**	**Intra-assay CV (%)^a^**				**Correlation Coefficient (r)**			**Inter-laboratory CV (%)^b^**
	**Lab 1**	**Lab 2**	**Lab 3**		**1 vs. 2**	**1 vs. 3**	**2 vs. 3**	
gp160-n	1.2	2.9	1.8		0.49	0.92	0.39	15.5
gp41-n	0.5	1.5	2.0		-0.05	0.60	-0.13	9.9
gp120-n	3.2	4.2	2.1		0.82	0.91	0.75	21.3
gp160-a	2.4	4.3	4.1		0.91	0.91	0.91	8.5
gp41-a	1.6	3.7	3.1		0.93	0.90	0.90	11.3
gp120-a	5.1	6.1	5.6		0.90	0.85	0.93	17.1

^a^Calculated based on intra-assay replicate samples.

^b^Median value for all 3 laboratories.

**Table 3:** Lot-to-lot comparison.

**Analyte**	**CV (%)**		
	**Lab 1**	**Lab 2**	**Lab 3**
gp160-n	10.6	11.3	17.4
gp41-n	5.0	13.1	11.2
gp120-n	6.7	18.4	12.5
gp160-a	5.5	4.8	5.2
gp41-a	14.1	9.7	6.3
gp120-a	12.7	16.0	6.4

**Figure 3:**
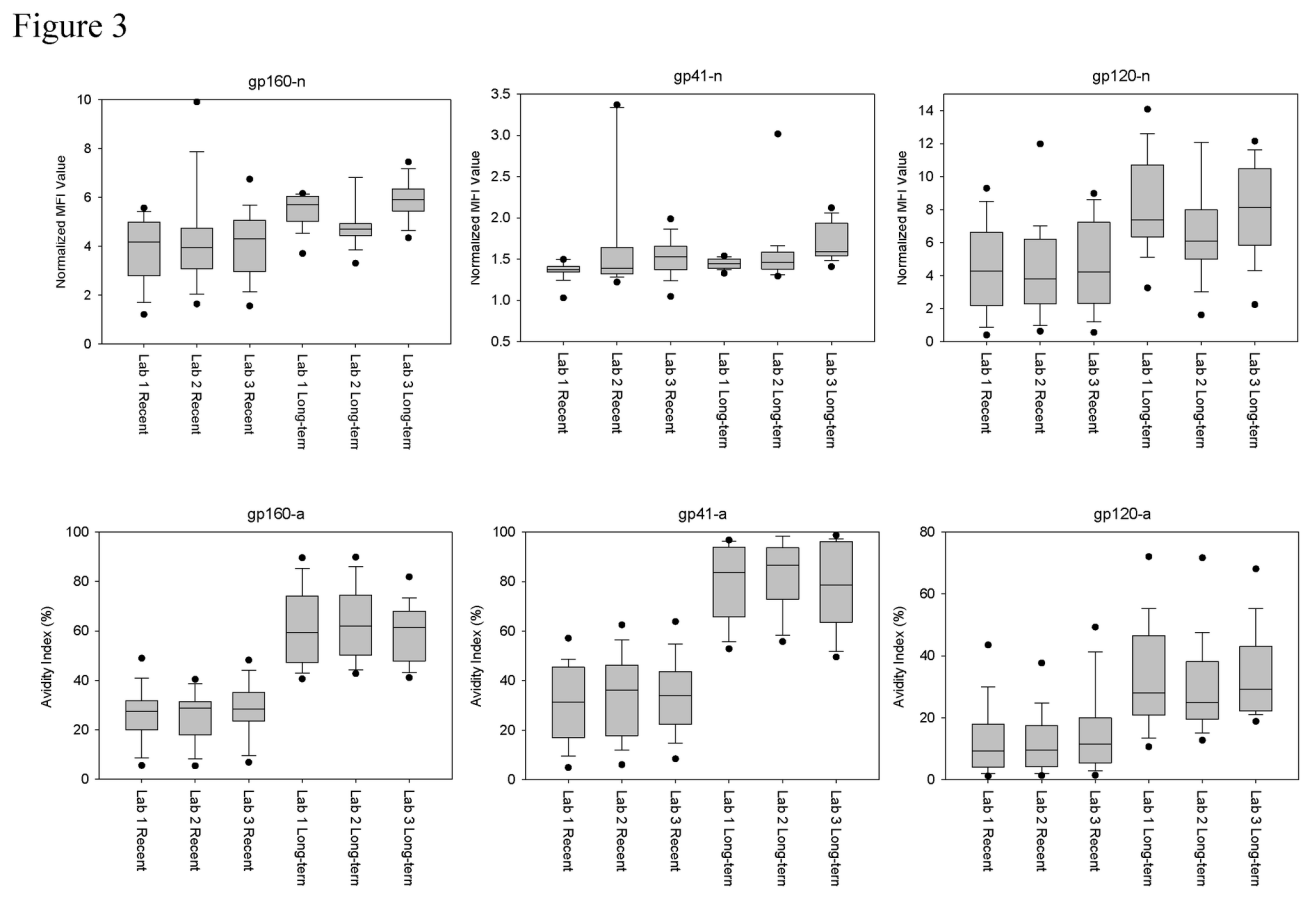
Inter-laboratory kit performance. The normalized MFI values (n) and avidity index (a) of known recent and long-term specimens, as measured by the prototype kits, were compared for each laboratory.

 Comparison of kit performance for the three laboratories with known recent and long-term specimens is shown in Figure 3. The distinction in reactivity between recent and long-term specimens was similar for all three laboratories, with the greatest separation in reactivity and inter-laboratory consistency observed for the avidity values. 

## Discussion

The HIV-1-specific Bio-Plex assay, described previously [11,26], allows for the measurement of HIV-specific antibody levels and avidity to multiple analytes for improved cross-sectional HIV-1 incidence estimates. Thus far, all of the assay components have been acquired or produced in-house and validation studies have been performed in a single laboratory. Although preliminary studies indicate that the in-house multiplex assay yields promise, the current format may not be practical for widespread dissemination and use. We have demonstrated the feasibility of producing a commercial kit with intra-assay and inter-operator reproducibility, amongst three separate laboratories.

Since the performance of the multiplex assay relies primarily on binding of antibodies to antigen-coupled microspheres, it is essential to demonstrate that microsphere sets and assay reagents can be produced outside the reference laboratory and yield reproducible results. Overall, consistent results were observed between the in-house assay and commercial kit, with longitudinal seroconversion panels. The calibrator is an important component of the assay as it controls for slight variations in MFI readings from run-to-run and may serve to normalize lot-to-lot differences with future commercial kits, as observed with the BED-CEIA assay [14]. Reactivity with the calibrator, however, appeared to affect the range in normalized MFI values observed for each analyte. The kit gp160-n and gp120-n exhibited a greater range of reactivity and approximately half the MFI value for the calibrator, as compared to the in-house assay, which is desirable for increased distinction in reactivity between samples. Since the HIV-1 recombinant proteins coupled to the microspheres were obtained from the same commercial source for both the in-house assay and kit, variations in reactivity may be due to lot-to-lot differences of the recombinant proteins or lab-specific coupling procedures. The calibrator and microsphere sets in the pilot kit will be further validated to ensure maximum separation in reactivity between samples from recent and long-term infections. Furthermore, the current calibrator was created in the reference laboratory by pooling plasma from HIV positive individuals and, therefore, has limited availability. For kit production, we will evaluate the possibility of commercial calibrator production. 

In the absence of validated assay cutoffs and mean durations of recency (MDRs), the kit performance is best determined by comparing the reactivity of well-characterized specimens from individuals with known recent and long-term infections. Despite slight differences in the normalized MFI value or avidity index scale between the in-house assay and the kit, there was minimal to no overlap in reactivity between known recent and long-term samples, with the exception of gp41-n, as described previously [11]. Although the current recombinant gp41 protein included in the assay does not provide discrimination based on antibody levels, due to relatively high reactivity with both recent and long-term samples, the avidity measures display a wide dynamic range and may serve as a more robust indicator of recent infection. We are currently evaluating several subtype-conserved recombinant gp41 proteins and peptides to identify an analyte that provides a measurable distinction in both antibody levels and avidity with specimens from individuals infected with diverse HIV-1 subtypes. One of the major benefits of the Bio-Plex format is the relative ease in which analytes can be added or removed from the assay, without affecting the remaining components or assay protocol.

 An inter-laboratory assessment of the prototype multiplex kits was performed to evaluate operator performance of the assay protocol and potential variation in assay results, which indicated acceptable concordance amongst the three participating laboratories and similar separation in reactivity between recent and long-term specimens. These results are encouraging, given that the reference laboratory distributed the assay protocol to all operators, but did not provide training on the assay to the test operators. One laboratory exhibited poor correlation with the other laboratories for gp160-n, although the median inter-laboratory CV remained within acceptable limits (Table 2). The divergent results could not be investigated further due to limited kit availability; however, this underscores the importance of operator training prior to initiation of future reproducibility studies. Inter-laboratory correlation for gp41-n was deceptively low due to a limited range of reactivity for the analyte, but the median CV indicated reproducible results. Overall, the avidity measures were robust and highly reproducible, which is not surprising given that avidity index is calculated relative to an untreated control on the same assay plate. 

For initial development and validation studies, all samples were tested in duplicate and the average MFI value of the replicates was used to calculate the normalized MFI value or avidity index. However, the low intra-assay CVs reported by all three laboratories indicate that reliable results may be provided with a single sample reading, which can reduce reagent cost. In the future assay format, a cutoff value will be selected for the IgG control bead set to identify sample output values that are invalid due to plating or machine reading error and would require repeat testing. Further assay refinements may include incorporation of subtype-conserved analytes, given that all recombinant proteins used in the current assay format are subtype-B derived. It is not known how the current format of the assay will perform with specimens from non-B subtype infections. Long-term stability studies will be needed to assess the shelf-life and storage temperature for the kit, as well as large scale inter-laboratory evaluations to assess operator acceptability and kit performance. 

 In summary, we have demonstrated the feasibility of producing a multiplex bead-based assay for measuring HIV antibody levels and avidity in kit form. Given the potential for improved incidence estimates based on intra-assay algorithms of multiple analyte measures, a commercial kit will allow for the transfer of technology to multiple and diverse laboratories for widespread evaluation and use of the assay. 
